# HIV risk behaviors and condom use in west region of Cameroon: need for HIV prevention strategies shifted towards HIV seropositive individuals

**DOI:** 10.1186/1471-2334-14-S2-P11

**Published:** 2014-05-23

**Authors:** Celine Nkenfou, Japhette T Kembou, Irenee Domkam, Appolinaire Djikeng, Timoleon Tchuinkam

**Affiliations:** 1 Chantal Biya International Research Center, Yaoundé, Cameroon

## Introduction

Implementation of HIV/AIDS control programs in Cameroon has not completely limited the spreading of the disease. Few studies conducted in West region of Cameroon determined factors favoring disease propagation; thus the need of an assessment so as to design strategies which will optimally limit the rate of transmission of HIV in this area.

## Materials and methods

Participants attending voluntary counseling during a screening campaign were recruited for a cross-sectional study after informed consent. Interview-administered questionnaires were used for data collection. HIV sero-status was assessed following the national algorithm and by oral fluid rapid test. Analyses of data were carried out using Microsoft Excel 2010 and the statistical package for social sciences (SPSS) at a level of significance of 5%.

## Results

66.7% of men and 61.1% of women declared scarification exposures. Poor knowledge of the disease was reported by 62.5% of men and 54.7% of women. They were less likely to use condoms (OR= 0.9; 95% CI: 0.6- 1.3). Multiple sexual partners were noted for 34.7% of men and 24.5% of women, who were found significantly less likely to use condoms (OR= 0.5; 95% CI: 0.3-0.7). 43.3% of patients declared occasional usage of condoms with women less likely to use them (OR= 0.8; 95% CI: 0.5-1.1). Unawareness on the serologic status of the sexual partner was also observed in 31.4% of men and 43.3% of women (95% CI: 0.5-1.2). HIV seropositive study participants used condoms occasionally (in 71.4% of cases) even though aware of their sero-status. They were not aware of the sero-status of their sexual partners in 57.1% of cases. Moreover 50% of them had more than one sexual partner. All these should be incorporated when designing prevention strategies to reduce disease impact.

## Conclusions

Rates of risky behaviors noted among HIV positive individuals should raise our attention on implementation of prevention strategies shifted towards this particular group of people.

